# Supplementary material for: Maternal Dietary Diversity and Birth Weight in Offspring: Evidence from a Chinese Population-Based Study
Source: Int J Environ Res Public Health. 2023 Feb 12;20(4):3228. doi: 10.3390/ijerph20043228 (PMC9960126; doi:10.3390/ijerph20043228)
Supplement: Supplementary file 1 [file ijerph-20-03228-s001.zip › ijerph-2180980-supplementary.pdf]

Supplementary files

Content

Table S1 The characteristics of the participants included and excluded

Table S2 Association between MDD-W score and neonatal birth weight by quantile regression (n=6805)

Table S3 Association between DDS-PCA and low birth weight by Logistic regression (*OR* (*95% CI*)) (n=6805)

Table S4 Association between MDD-W score and low birth weight in the participants with children aged <12 months (n=5057)

Table S5 Association between MDD-W score and low birth weight in the participants with complete values of all covariates (n=5876)

Figure S1 Subgroup analysis on association of MDD-W score and low birth weight by linear regression

Table S1 The characteristics of the participants included and excluded

	Participants*		*P value*
	Included (n_1=6805)	Exclude (n_2=1404)	
Maternal age (year), $\bar{x} \pm s$	26.14±4.62	25.96±4.61	0.212
Gestational weeks (week), $\bar{x} \pm s$	39.56±1.33	39.52±1.61	0.339
Neonate birth weight (g) , $\bar{x} \pm s$	3267.79±449.32	3271.16±478.94	0.172
Neonate gender, n (%)			
male	3580 (53.4)	645 (53.1)	0.856
female	3128 (46.6)	570 (46.9)	
Mean monthly household expenditure (Yuan)	2338.78±1533.77	2265.19±1851.99	**0.021**
Maternal registered permanent residence, n (%)			
urban	991 (14.6)	132 (10.8)	**<0.001**
rural	5776 (85.4)	1085 (89.2)	
Antenatal examination			
yes	6707 (98.9)	1201 (98.1)	**0.020**
no	74 (1.1)	23 (1.9)	
Folic acid supplements, n (%)			
yes	5006 (73.9)	841 (68.7)	**<0.001**
no	1768 (26.1)	384 (31.3)	
Illness during pregnancy, n (%)			
yes	4000 (58.9)	624 (50.6)	**<0.001**
no	2788 (41.1)	609 (49.4)	
Maternal education level, n (%)			
low	619 (9.1)	123 (10.0)	0.556
middle	3676 (54.2)	651 (53.0)	
high	2493 (36.7)	454 (37.0)	
Maternal career, n (%)			
farmers	4866 (72.0)	892 (72.8)	0.852
workers and merchants	925 (13.7)	162 (13.2)	
intellectuals	963 (14.3)	171 (14.0)	

*There were slight missing values on variables of maternal age, gestational weeks, neonate birth weight, neonate gender, household expenditure maternal registered permanent residence, antenatal examination, folic acid supplements, illness during pregnancy, maternal education level and maternal career.

Table S2 Association between MDD-W score and neonatal birth weight by quantile regression (n=6805)

	Newborn birth weight								
	**q=0.1**	**q=0.2**	**q=0.3**	**q=0.4**	**q=0.5**	**q=0.6**	**q=0.7**	**q=0.8**	**q=0.9**
MDD-W	0.65	4.17	5.96	4.98	7.09	6.86	5.00	4.82	1.40
	(-8.42, 9.72)	(-2.48, 10.82)	(-0.23, 12.15)	(-1.28, 11.24)	(0.82, 13.37)	(0.27, 13.46)	(-1.98, 11.98)	(-2.99, 12.63)	(-7.76, 10.57)
P value	0.888	0.219	0.059	0.119	**0.027**	**0.041**	0.160	0.226	0.764
Animal-based food	17.37	18.34	13.59	8.33	7.14	4.67	5.15	5.33	0.80
	(-1.29, 36.03)	(4.73, 31.96)	(0.87, 26.31)	(-4.62, 21.29)	(-6.12, 20.40)	(-9.10, 18.43)	(-9.10, 19.40)	(-10.89, 21.55)	(-18.36. 19.95)
P value	0.068	**0.008**	**0.036**	0.207	0.291	0.506	0.479	0.519	0.935
Non Animal-based food	-2.38	-1.16	4.60	6.25	9.12	10.42	7.31	6.01	2.54
	(-14.31, 9.56)	(-9.92, 7.60)	(-3.62, 12.82)	(-2.03, 14.53)	(0.74, 17.50)	(1.68, 19.15)	(-1.82, 16.43)	(-4.28, 16.30)	(-9.74, 14.82)
P value	0.696	0.795	0.273	0.139	**0.033**	**0.019**	0.117	0.252	0.685
Ratio	63.44	82.72	32.78	9.42	＜0.01	-30.76	＜0.01	-8.43	-48.18
	(-3.38, 130.26)	(32.90, 132.55)	(-14.47, 80.02)	(-38.69, 57.54)	(-48.89, 48.89)	(-81.01, 19.50)	(-52.15, 52.15)	(-68.10, 51.23)	(-116.77, 20.41)
P value	0.063	**0.001**	0.174	0.701	1.000	0.230	1.000	0.782	0.169

Note: Adjusted for maternal age, gestational weeks, neonate gender, folic acid supplements, family economic status, maternal registered permanent residence, antenatal examination level, illness during pregnancy, maternal education level and maternal career. Ratio: ratio of animal-based food DDS to non animal-based food DDS.

Table S3 Association between DDS-PCA and low birth weight by Logistic regression (*OR* (*95% CI*)) (n=6805)

	DDS-PCA		*P*	DDS-PCA tertiles			*P for trend*
	OR	*95%CI*		*T1*	*T2*	*T3*	
**Model 1**							
	0.61	0.47, 0.80	**<0.001**	1.00	0.68 (0.49, 0.93)	0.69 (0.50, 0.95)	**0.022**
**Model 2**							
	0.59	0.44, 0.77	**<0.001**	1.00	0.72 (0.51, 1.01)	0.66 (0.47, 0.94)	**0.020**
**Model 3**							
	0.59	0.45, 0.78	**<0.001**	1.00	0.71 (0.50, 1.01)	0.68 (0.48, 0.96)	**0.027**

Note: Model 1 was unadjusted. Model 2 was adjusted for maternal age, gestational weeks, neonate gender and folic acid supplements. Model 3 was further adjusted for family economic status, maternal registered permanent residence, antenatal examination level, illness during pregnancy, maternal education level and maternal career.

Table S4 Association between MDD-W score and low birth weight in the participants with children aged <12 months (n=5057)

	MDD-W		*P*	MDD-W tertiles			*P for trend*
	OR	*95%CI*		*T1*	*T2*	*T3*	
MDD-W	0.91	0.83, 0.99	**0.035**	1.00	0.60 (0.41, 0.89)	0.56 (0.37, 0.86)	**0.005**
Animal-based food	0.72	0.59, 0.88	**0.001**	1.00	0.53 (0.36, 0.78)	0.64 (0.38, 1.08)	**0.011**
Non Animal-based food	0.96	0.86, 1.08	0.515	1.00	0.90 (0.59, 1.36)	0.84 (0.57, 1.25)	0.384
Ratio	0.28	0.11, 0.68	**0.005**	1.00	0.75 (0.51, 1.11)	0.57 (0.38, 0.88)	**0.009**

Note: Adjusted for maternal age, gestational weeks, neonate gender, folic acid supplements, family economic status, maternal registered permanent residence, antenatal examination level, illness during pregnancy, maternal education level and maternal career. Ratio: ratio of animal-based food DDS to non animal-based food DDS.

Table S5 Association between MDD-W score and low birth weight in the participants with complete values of all covariates (n=5876)

	MDD-W		*P*	MDD-W tertiles			*P for trend*
	OR	*95%CI*		*T1*	*T2*	*T3*	
MDD-W	0.89	0.82, 0.97	**0.009**	1.00	0.56 (0.39, 0.81)	0.63 (0.42, 0.93)	**0.010**
Animal-based food	0.72	0.61, 0.87	**<0.001**	1.00	0.55 (0.39, 0.79)	0.58 (0.35, 0.99)	**0.004**
Non Animal-based food	0.93	0.84, 1.04	0.197	1.00	0.79 (0.54, 1.18)	0.86 (0.60, 1.23)	0.361
Ratio	0.30	0.14, 0.65	**0.002**	1.00	0.78 (0.54, 1.11)	0.58 (0.38, 0.87)	**0.007**

Note: Adjusted for maternal age, gestational weeks, neonate gender, folic acid supplements, family economic status, maternal registered permanent residence, antenatal examination level, illness during pregnancy, maternal education level and maternal career. Ratio: ratio of animal-based food DDS to non animal-based food DDS.

Group	OR (95%CI)	P value
**Neonate gender**		
male	0.91 (0.81, 1.03)	0.127
female	0.91 (0.82, 1.00)	0.051
**Maternal age (year)**		
≤20	0.93 (0.68, 1.26)	0.628
＞20, ≤30	0.88 (0.81, 0.96)	0.004
＞30	1.07 (0.89, 1.30)	0.468
**Maternal registered permanent residence**		
urban	1.02 (0.80, 1.30)	0.883
rural	0.91 (0.84, 0.98)	0.017
**Folic acid supplements**		
yes	0.91 (0.83, 0.99)	0.037
no	0.94 (0.82, 1.07)	0.342
**Antenatal examination level**		
low	0.86 (0.75, 0.98)	0.019
middle	0.85 (0.73, 0.99)	0.036
high	1.00 (0.88, 1.14)	0.950
**Illness during pregnancy**		
yes	0.91 (0.82, 1.00)	0.054
no	0.91 (0.81, 1.02)	0.108
**Family economic status**		
low	0.89 (0.79, 1.01)	0.081
middle	0.95 (0.84, 1.08)	0.443
high	0.87 (0.76, 1.01)	0.064
**Maternal education level**		
low	0.96 (0.64, 1.44)	0.843
middle	0.91 (0.83, 1.00)	0.050
high	0.90 (0.78, 1.03)	0.135
**Maternal career**		
farmers	0.90 (0.82, 0.98)	0.020
workers and merchants	0.92 (0.75, 1.13)	0.436
intellectuals	1.00 (0.79, 1.26)	0.983
**Total**	0.91 (0.85, 0.98)	0.015

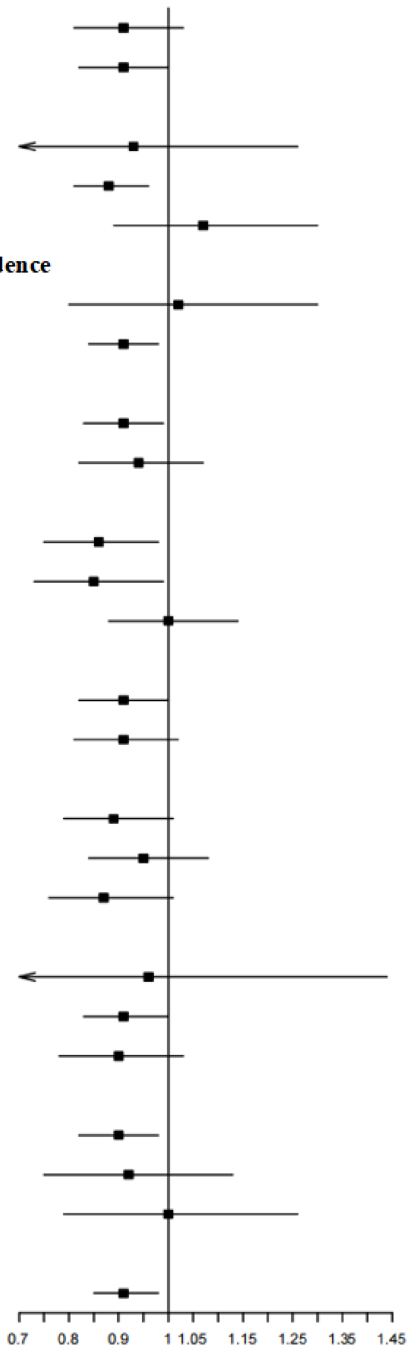

Figure S1 Subgroup analysis on association of MDD-W score and low birth weight by linear regression

Note: analysis was adjusted for maternal age, gestational weeks, neonate gender, folic acid supplements, family economic status, maternal registered permanent residence, antenatal examination level, illness during pregnancy, maternal education level and maternal career when necessary.
